# 伴嗜酸性粒细胞增多和FGFR1重排髓系肿瘤1例

**DOI:** 10.3760/cma.j.issn.0253-2727.2022.01.018

**Published:** 2022-01

**Authors:** 昱瑛 李, 晨 王, 明 张, 忠华 杜, 瑞萍 胡, 晶 白, 克举 苏, 晓亮 刘, 岩 杨, 业辉 谭, 素君 高, 薇 李

**Affiliations:** 吉林大学第一医院肿瘤中心血液科，长春 130021 Department of Hematology, Tumor Center, the First Hospital of Jilin University, Changchun 130012, China

患者，男性，56岁，因“发热半个月，腹胀7 d”于2019年5月入住吉林大学第一医院血液科。否认皮肤瘙痒，咳嗽、咳痰，腹痛、腹泻等胃肠道症状。既往糖尿病病史8年，血糖控制可；有土霉素过敏史；吸烟史20余年（已戒20年），饮酒史30年；近期无旅游史，无不洁饮食，未食用生冷海鲜食物。查体：ECOG评分3分，一般状态差，无贫血貌，皮肤及巩膜黄染，全身浅表淋巴结未触及，胸骨压痛阴性，双肺底呼吸音弱，心脏查体无明显异常，腹部膨隆，肝脾肋缘下未触及，移动性浊音阳性，双下肢无水肿。血常规：WBC 66.6×10^9^/L，嗜酸性粒细胞22.25％（14.82×10^9^/L），HGB 158 g/L，PLT 20×10^9^/L；肝功能：谷丙转氨酶39 U/L，谷草转氨酶48 U/L，总胆红素73 µmol/L，直接胆红素39 µmol/L，白蛋白26 g/L；呼吸道病毒、巨细胞病毒（CMV）、EB病毒（EBV）、降钙素原（PCT）等感染相关检验阴性，自身免疫系统疾病筛查阴性。浅表淋巴结超声可见颈部、腋下及腹股沟多发淋巴结肿大（最大1.3 cm×0.6 cm，皮髓结构尚清）。CT示“双侧胸腔及腹腔中等量积液，肝脏脾脏不大，腹主动脉旁、腹腔内见多发淋巴结影（0.4～1.4 cm）”。骨髓象：有核细胞增生明显活跃，粒系增生明显活跃，原始粒细胞比例增高（0.175），嗜酸细胞比例明显增高（0.325），其中幼稚嗜酸性粒细胞占0.065，粒细胞颗粒明显增多；红系增生活跃；淋巴细胞比例减低，形态正常。骨髓活检病理：骨髓有核细胞增生极度活跃，粒红比例增高，幼稚细胞易见，嗜酸细胞多见，网状纤维染色MF-2级灶性。骨髓免疫分型：异常髓系原始细胞占有核细胞4.52％，主要表达CD34^str^、CD33、HLA-DR、CD123、CD7、CD11b^dim^、CD25，嗜酸性粒细胞比例明显增高（占28.1％）。髓系/淋系肿瘤融合基因筛查：BCR-ABL1等32种融合基因阴性。二代基因测序未检出RUNX1、DNMT3A、TET2等111种髓系/淋系肿瘤相关基因突变。荧光原位杂交（FISH）检测：FGFR1基因重排占94.5％（图1），PDGFRα、PDGFRβ及JAK2基因重排阴性。染色体核型46,XY,-7,t（8;13）（p11.2;q12）, + der（13）t（8;13）（p11.2;q12）（?）[18]/46,XY, t（8;13）（p11.2;q12）[2]。RT-PCR及直接测序证实ZMYM2-FGFR1融合基因阳性。临床诊断：伴嗜酸性粒细胞增多和FGFR1重排的髓系肿瘤。

**图1:**
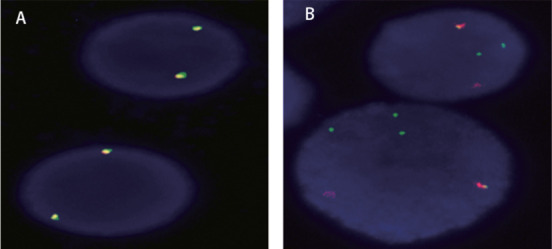
荧光原位杂交（FISH）检测FGFR1 基因重排阳性 A：双色FISH检测正常细胞，显示为红绿融合信号；B：双色FISH检测患者细胞，可见1R1G1F（R为红色信号标记5′FGFR1，G为绿色信号标记3′FGFR1，F为融合信号）即FGFR1基因重排阳性

2019年6月予去甲氧柔红霉素（10 mg/m^2^，第1、2天）联合地西他滨（20 mg/m^2^，第1～5天）化疗，患者发热、腹胀症状迅速缓解，浆膜腔积液消失，外周血白细胞计数及嗜酸性粒细胞比例下降（化疗结束第10天骨髓嗜酸性粒细胞10％，原始粒细胞比例0％）。化疗结束第22天，患者再次出现发热，血常规白细胞及嗜酸性粒细胞比例升高，骨髓嗜酸性粒细胞0.220，原始粒细胞比例0.015，考虑治疗无效。于2019年7月行单倍型造血干细胞移植（子供父），预处理方案为地西他滨+改良BU/CY（白消安/环磷酰胺）+抗胸腺细胞球蛋白（ATG），回输供者CD34^+^细胞3.11×10^6^/kg，单个核细胞6.30×10^8^/kg。移植后14 d达细胞遗传学缓解，180 d达分子生物学缓解。随访至移植后18个月，仍为分子生物学缓解。

